# Does Participating in Physical Exercise Make Chinese Residents Happier?—Empirical Research Based on 2018 Chinese General Social Survey

**DOI:** 10.3390/ijerph191912732

**Published:** 2022-10-05

**Authors:** Liluo Gan, Yumei Jiang

**Affiliations:** School of Physical Education, Huazhong University of Science and Technology, Wuhan 430074, China

**Keywords:** physical exercise, subjective well-being, OLS model, propensity score matching

## Abstract

Participating in physical exercise to improve fitness, as well as experience the social and economic functions of sport, can help individuals improve their subjective well-being. The ordinary least squares (OLS) regression model was used to empirically study the impact of the frequency and intensity of physical exercise on Chinese residents’ subjective well-being and its mechanisms using data from the 2018 China General Social Survey (CGSS). The findings revealed that participating in physical exercise significantly increased Chinese residents’ subjective well-being; the impact of physical exercise on Chinese residents’ subjective well-being varied with age, marriage, political status, and so on, and participation in physical exercise improved in rural individuals, male individuals, and individuals from the eastern regions. Higher intensity physical exercise increases the likelihood of subjective well-being; urban individuals, female individuals, and individuals in the central and eastern regions have a higher probability of improving subjective well-being.

## 1. Introduction

According to the report of the Communist Party of China’s 19th National Congress, “adhering to the people-centered approach is the fundamental position of upholding and developing socialism with Chinese characteristics in the new era” [1]. Physical exercise has been prioritized and developed as part of the dual promotion of policy guidance and practical needs for national fitness and happiness, which has improved people’s quality of life.

Physical exercise is the main measure for “Healthy China 2030” as a way to improve Chinese residents’ health and quality of life. Most research on the link between exercise and happiness focuses on reducing negative emotions through exercise [2,3] (i.e., reducing anxiety, depression, loneliness, stress, and sleep quality). Physical exercise can provide psychological pleasure and reduce negative emotions, but it does not guarantee happiness [4]. Can exercise boost Chinese residents’ well-being? This study builds on a sociological empirical foundation from the perspective of physical exercise to investigate the relationship between physical exercise and subjective well-being to better guide people in improving their quality of life and life satisfaction through physical exercise, as well as their subjective well-being.

According to the World Health Organization, physical activity is “Any bodily movement produced by skeletal muscles that requires energy expenditure.” The topic of this article is physical exercise. Physical exercise is the process of exercising the body using various physical means in conjunction with natural forces and engaging in physical activities to improve health and physical fitness [5]. Participating in physical exercise, as defined in this article, is distinct from competitive sports and school sports, and primarily refers to physical activities in which Chinese residents engage in order to improve their health and physical fitness through the use of a specific sport.

Cohen defines subjective well-being as a person’s subjective and holistic assessment of their living conditions [6]. Diener et al. combined two views. Subjective well-being is people’s overall evaluation of their own quality of life according to internal standards. It is a comprehensive evaluation of people’s satisfaction with life and its various aspects, predominantly concerning happy emotions [7]. Subjective well-being emphasizes an individual’s overall assessment of life quality relative to self-determined standards [8]. Early theories of subjective well-being focused on how events and situations affect it [9,10,11]. The earliest research in this area dates back to Jacobson’s study on the intervention of anxiety with a specific form of motor exercise, which can be considered a study of physical exercise and the affective dimension of subjective well-being.

Some studies find that an individual’s internal construction determines how external factors are perceived, influencing well-being [12]. Scholars began focusing on internal factors that affect subjective well-being, generalizing comparative theory, self-determination theory, and other theories. According to self-determination theory [13], every individual has a developmental need. If that need is met, the individual will develop toward healthy and optimal choices and experience the happiness of an active life.

According to some scholars, Chinese residents’ subjective well-being stems from a variety of factors, including exercise, which is a source of happiness [14]. Many scholars acknowledge exercise’s role in promoting mental health [15]. Petruzello analyzed 104 studies on the control effect of physical exercise on anxiety between 1960 and 1989 [16]. Both long-term and one-time aerobic exercises reduced state anxiety. Yazicioglu found in an experiment that people who exercised had a higher quality of life and life satisfaction [17]. The degree of physical exercise participation affects subjective well-being, and the more often someone exercises, the higher their subjective well-being will be.

Walking and jogging, soccer, volleyball, or gymnastics combined with relaxation exercises were studied by Bosscher [18]. Patients in the jogging or walking group reported significant reductions in depression and physical symptoms, as well as increased self-esteem and significantly better physical status. Patients in the mixed group, on the other hand, showed no physical or psychological changes. According to these studies, the psychological benefits of exercise vary greatly depending on physical exercise elements such as exercise intensity.

Jogging or walking reduced depression and physical symptoms, increased self-esteem, and improved physical status. Mixed-group patients showed no physical or psychological changes. According to these studies, the psychological benefits of exercise depend on exercise intensity.

Previous studies examined a single variable, such as exercise type, intensity, frequency, and duration, rather than their combined effects and interactions on subjective well-being. This study proposes the following hypotheses.

**Hypothesis** **1:** *Frequent physical exercise would have a significant positive effect on the subjective well-being of Chinese residents*.

**Hypothesis** **2:** *Intense physical exercise would have a significant positive effect on the subjective well-being of Chinese residents*.

The purpose of this study is to determine whether physical exercise can improve the subjective well-being of Chinese residents and to explain the causal relationship between the two. The data from the China General Social Survey 2018 (CGSS2018) are being used to strengthen the authority and breadth of this research sample. The OLS regression model was used to empirically study the impact of physical exercise on individual subjective well-being, as well as to investigate the heterogeneity of physical exercise to improve subjective well-being based on gender, regional, and urban–rural differences. The study used the “propensity score matching” method to obtain more robust estimation results in order to overcome sample selection bias and reveal the causal relationship between the two.

## 2. Materials and Methods

### 2.1. Materials

The 2018 Chinese General Social Survey was used for this study (CGSS). Renmin University of China is conducting the country’s first ever large-scale social survey, and they are completing it at their China Survey and Data Center. Using consistent and systematic data collection on the Chinese people and various aspects of Chinese society, the survey aims to provide a summary of long-term trends in social change. Over the course of 2018, 12,787 valid samples were collected as part of the CGSS questionnaire survey. This comprehensive and methodical survey included interviews with over 15,000 people from all walks of life and was conducted in 125 counties across 28 provinces, autonomous regions, and municipalities in China. After removing individual samples with missing values and extreme outliers, 8445 valid full samples were used to analyze the variables necessary for the subjects involved in subjective well-being and physical exercise.

### 2.2. Variable Selection

In this paper, the explained variable is subjective well-being. Khalek et al. assessed happiness by asking a single question: “Are you generally happy?” [19]. It is reliable, valid, and feasible to measure well-being using a single item. Based on this, this paper uses the results of the China General Social Survey (CGSS) happiness survey to reflect Chinese residents’ happiness. The source item is A36: “Do you think your life is generally happy?”

The core explanatory variables are the frequency and intensity of physical exercise. A309 is the source item. “Do you engage in physical exercise?” is the question. “Every day, several times a week, several times a month, several times a year, or less,” are the answers. Assign a value of 1 for participating in physical exercise and a value of 0 for not participating in physical exercise to the answer “never”. The source item for physical exercise intensity is A15a: “How many times per week did you typically perform 30 minutes of physical exercise that caused you to sweat in the previous 12 months?”.

To reduce the possibility of model estimation bias, this study analyzes control variables from three levels: individual, family, and society (see Table 1). Gender, age, ethnicity, religious belief, education level, personal income, political status, marital status, household registration status, and working hours are examples of individual-level control variables. Control variables at the household level include total household income, household size, and household economic status. Location, social interaction, socioeconomic status, and social medical security are all control variables at the social level.

### 2.3. Analysis

#### 2.3.1. OLS Regression

The subjective well-being of Chinese residents is used as the explained variable in this study. The subjective well-being score is usually treated as a continuous variable in research on subjective well-being at home and abroad, and the OLS model can more intuitively show the impact of various factors on subjective well-being. As a result, this paper employs the OLS model to investigate the effect of physical exercise on subjective well-being and develops a multiple regression model (1), as follows:

happiness_i_ = α_0_ + α_1_EF_i_ + α_2_EI_i_ + ∑β_i_X_i_ + ε_i_ (1)

happiness_i_ is the subjective well-being of the ith subject, EF is exercise frequency, EI is exercise intensity, X is other control variables affecting subjective well-being, and α and β are the corresponding regression coefficients.

#### 2.3.2. Grouped Regression

The influence of physical exercise involvement on the subjective well-being of Chinese inhabitants may vary based on the specific characteristics of Chinese residents. As a result, we ran a grouped regression analysis on the sample data, which was divided into gender, region of residence, and urban/rural area.

#### 2.3.3. Propensity Score Matching

To increase the reliability of the aforementioned regression results by reducing the difficulties of sample selection bias and omitted variables. First, OLS regression models were used to evaluate the probability of positive or negative impacts of physical exercise on the subjective well-being of Chinese citizens. The variables were then matched based on the propensity score values, and the standardized bias of each variable for the matched treatment and control groups was determined to eliminate the model’s selection bias and make the treatment group “similar” to the control group. The data are well balanced if the matching results (in the case of nearest neighbor matching) reveal that the standard deviation of all samples is less than 10%. Finally, the nearest neighbor matching, radius matching, and kernel matching approaches were used to calculate the mean treatment effects of physical exercise on the subjective well-being of Chinese residents.

## 3. Results

### 3.1. Population Sample Estimation Results and Analysis

To test the impact of physical exercise on improving subjective well-being, OLS regression was performed using model (1), with subjective well-being as the explained variable, the explanatory variable, and the control variables included. Given that the cross-sectional data used in this study may have heteroscedasticity issues, the least squares method was used to regress the model, but robust standard errors were used in the regression. The statistical theory demonstrates that even when there is heteroscedasticity in a large sample, using OLS regression with robust standard error results in a consistent estimation of regression coefficients, and parameter estimation and hypothesis testing can be performed as usual [20].

Table 2 uses stepwise regression to assess the impact of physical exercise on Chinese residents’ subjective well-being. When other variables are not controlled in Model (1) in Table 2, the estimated coefficients of physical exercise frequency and intensity are both positive and pass the 1% significance test. Increasing physical exercise frequency and intensity can improve Chinese residents’ subjective well-being. Physical exercise improves people’s quality of life and subjective well-being, supporting hypotheses 1 and 2. The Garatachea study found that exercise improves subjective well-being.

Model (2) in Table 2’s second column includes individual-level control variables. Women have higher subjective well-being than men, according to the gender coefficient. Middle-aged people have lower subjective well-being than young and old people. At 60, a person’s contentment and happiness improve significantly, reflecting the main labor force’s age distribution. Middle-aged people experience life pressures, social responsibilities, and a “mid-life crisis”.

Ethnic minority Chinese residents have higher subjective well-being than Han Chinese residents, but this fails the 10% significance test. The estimated religious belief coefficient is significantly negative, indicating that Chinese who believe in God have higher subjective well-being. Higher education levels are associated with higher subjective well-being in China. Personal income’s estimated coefficient is also positive, indicating that higher-income Chinese residents have higher subjective well-being.

The estimated political affiliation coefficient is significantly positive, indicating CCP members have higher subjective well-being than non-members. The estimated marital status coefficient is positive, indicating married Chinese are happier than unmarried. The positive correlation between household registration, working hours, and subjective well-being fails the 10% significance test.

Subjective well-being is also related to family background, but the degree varies greatly. Model 3 in Table 2 includes household-level control variables. All estimated coefficients for family income, family population, and family economic status are significantly positive, indicating that Chinese residents with higher family income, larger families, and better economic status have higher subjective well-being. Peltzer surveyed students in 25 Asian, African, and American universities. Both positive and negative health behaviors were linked to subjective well-being, but college students from higher-income families had higher subjective well-being [21].

Socioeconomic status is a person’s social and economic standing. Family income, social network, and reputation all indicate socioeconomic status. In Table 2, Model 4 includes social control variables. Social interaction and socioeconomic status both had significantly positive coefficients, indicating that Chinese residents with higher socioeconomic status and more social interaction were happier. Location, social medical insurance, and subjective well-being correlated positively, but failed the 10% significance test. According to Gorely, adolescents with a higher socioeconomic status experience greater well-being when participating in physical activities [22].

### 3.2. Grouped Regression Analysis

This paper investigates the impact of exercise on the subjective well-being of Chinese residents of different genders, regions, and urban and rural areas. Table 3 shows the results. Regular exercise improves the subjective well-being of urban, eastern, and male Chinese residents (Table 3). Physical exercise improves the subjective well-being of Chinese residents in the central and eastern regions, as well as women, but has no effect on urban and rural residents.

#### 3.2.1. Analysis of Urban–Rural Heterogeneity

Table 3 shows that the regression coefficients of the intensity of physical exercise participation on the subjective well-being of urban and rural Chinese residents are both positive, the urban regression coefficient is greater than that of rural areas, and the 1% statistical level is significant, indicating that participating in physical exercise can effectively promote the subjective well-being of urban and rural Chinese residents. China’s unbalanced and insufficient development has been hampered by rural areas. “Hundreds of Millions of Farmers’ Fitness Activities” have addressed the lack of rural sports infrastructure through public welfare sports activities. Inadequate rural sports services only treat symptoms, not causes [23]. 

China’s current rural sports development relies primarily on government supply, has weakened support funds, and has an unsound operation mechanism and a disjointed supply and demand mechanism. Rural public sports services lack professional human resources, and sports social organizations are few and weak. The hollowing out and empty nesting phenomena are serious, but the village committee views public sports service governance as a minor issue [24]. These practical disadvantages limit rural public sports services’ ability to perform their essential role and cannot improve rural Chinese residents’ subjective well-being [25].

#### 3.2.2. Regional Heterogeneity Analysis

Table 3 shows that the frequency and intensity of Chinese residents’ physical exercise participation in the eastern region are both positively and significantly related to their subjective well-being, whereas only the intensity of Chinese residents’ physical exercise participation in the central region is both positively and significantly related to their subjective well-being, and the regression coefficient in the central region is greater than that in the eastern region. The frequency and intensity of physical exercise participation among Chinese residents in the western region, as well as the regression coefficient of subjective well-being, are both positive but not statistically significant, indicating that physical exercise has little impact on the subjective well-being of Chinese residents in the western region.

Because the eastern and central regions outperform the western regions in terms of economy, environment, income, and development of public sports services, when people’s material needs are met, greater emphasis is placed on spiritual and cultural life [26]. With the continuous improvement and rapid development of the public service systems of the provinces and cities along the Yangtze River Economic Belt in the central region in recent years, the level of public sports services has steadily improved, allowing people to have a more direct and tangible sense of happiness [27]. As a result, the regression coefficient shows that the central region’s coefficient of participation in physical exercise on subjective well-being is greater than that of the eastern region.

The western region’s economic development is relatively backward, and material resources are relatively scarce; material wealth can improve the happiness of Chinese residents. At the same time, when people are eagerly pursuing material wealth, they may be unwilling to invest time and money in physical exercise due to the influence of the concept of sports consumption. As a result, the impact of physical exercise on the subjective well-being of Chinese residents in the western region is unclear.

#### 3.2.3. Propensity Score Matching

**Figure 1:**
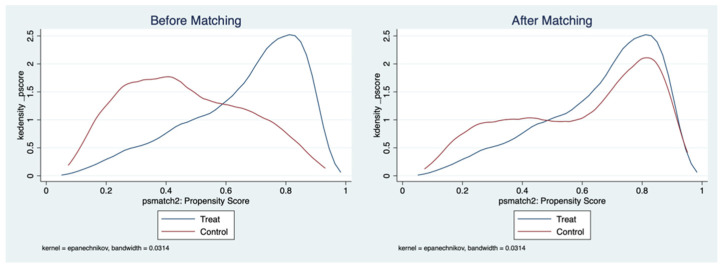
Kernel density distribution.

This study will use propensity score matching (PSM) to overcome the sample selection problem and solve the estimation bias in order to solve the endogeneity problem. To begin, we created a matching impact diagram for the kernel matching method to validate the rationality and significance of PSM. The kernel density distribution of the two groups of propensity score scores before and after matching is depicted in Figure 1. 

The figure shows that before the matching, the distribution of the propensity score for physical exercise in the treatment group and the control group showed obvious differences, and after the matching, the difference in the distribution of the propensity score for subjective well-being between the two groups was significantly reduced and the curves of the treatment group and the control group were almost the same, indicating that the matching impact was significant.

Before using propensity score matching (PSM), we must consider two points: whether the distribution of related variables in the treatment and control groups after matching is balanced, and whether the matching scores of the treatment and control groups overlap, that is, whether the propensity score matching method’s two assumptions of independence and joint support are met. As a result, the T-test was used first to determine whether there was a significant difference between the covariates of the two groups before and after matching. Table 4 displays the test results. All of the variables of the Chinese residents in the treatment and control groups existed or partially existed prior to the matching. The bias ratios were all less than 5% after matching, and there was no significant difference between the two groups of variables.

The deviation of most covariate means decreased after using the nearest neighbor matching method with one-to-one replacement, the standard deviation between the experimental and control groups was within 10%, and there was no significant covariate between the two groups. The matching impact test in Table 5 passed the LR test, the *p* value was 0.906, and the pseudo R2 value after matching decreased, indicating that the matching method under each covariate satisfied the conditional independence hypothesis. The equilibrium impact is positive, and the analysis results are trustworthy.

**Figure 2:**
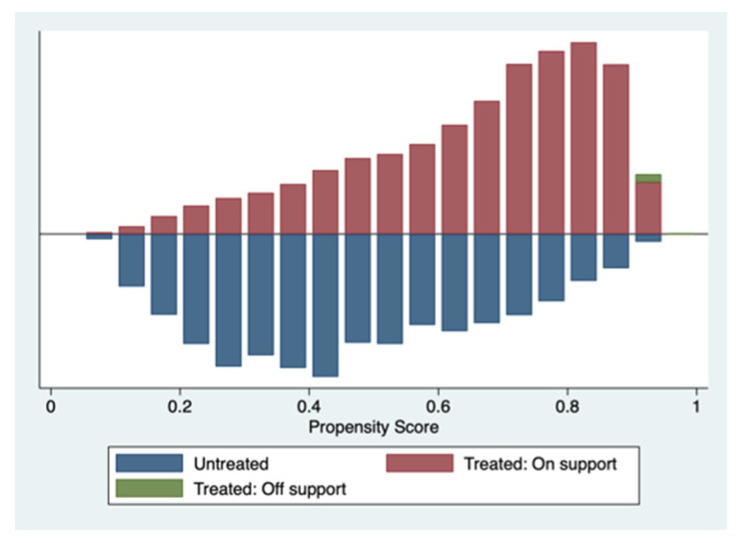
Common value range.

As shown in Table 5, there is no significant difference between the experimental and control groups in each covariate after using different matching methods, and both pass the LR test with a *p* value greater than 0.900, satisfying the conditional independence. Again, the matching quality is higher under the four matching methods tested in this study. Furthermore, as shown in Figure 2, the distribution of estimated propensity scores between the experimental and control groups is different, and most of the observed values are within the common value range. This satisfies the common support assumption, and only loses a small number of samples during matching estimation, demonstrating that the matching quality of this method is high.

## 4. Discussion

This research will employ the following three methods to learn if physical exercise does indeed boost the subjective well-being of Chinese residents. We first used OLS regression to analyze whether or not there is a correlation between exercise and happiness levels among Chinese citizens. Second, we used group regression to analyze how exercise affects people’s moods in various demographics. Finally, propensity score matching is used to examine the causal effects of exercise on the subjective well-being of Chinese citizens. Following these three procedures, we test the proposed hypothesis that physical exercise can make Chinese residents happier.

First, this study discovered a positive relationship between physical exercise and Chinese residents’ subjective well-being, implying that as Chinese residents’ participation in physical exercise increases in frequency and intensity, so does their subjective well-being. On the one hand, physical exercise can generate positive emotions, enhance the psychological experience, and improve mental health; people’s lifestyles and healthy behaviors will also change, resulting in improved physical health and subjective well-being. Physical exercise, on the other hand, can promote team friendship, establish a good social network relationship, and improve the level of social life quality and subjective well-being. Downward et al. discovered that sports based on social interaction can increase sports participants’ subjective well-being [9].

Second, we aimed to determine whether physical exercise improves the subjective well-being of Chinese residents with diverse characteristics. This study looks at gender differences, regional differences, and urban–rural differences. (1) Gender distinctions: The frequency of physical exercise improved male Chinese residents’ subjective well-being significantly, while the intensity of physical exercise improved female Chinese residents’ subjective well-being significantly. (2) Regional variations: Physical exercise can improve the subjective well-being of Chinese residents in the east and central regions, but not in the west. Sedlarski discovered that socioeconomic status, relative income, and consumption level all influence subjective well-being [28]. (3) Urban–rural disparities: For analysis and comparison, the research objects are divided into urban and rural areas, and it is discovered that physical exercise can promote the subjective well-being of both urban and rural Chinese residents, with Chinese residents who exercise in urban areas having higher subjective well-being. This conclusion holds up when examined again using the “propensity score matching” method.

## 5. Conclusions

The 2018 China General Social Survey was used to investigate the relationship between physical exercise and residents of China’s subjective well-being. OLS regression and propensity score matching were the statistical methods that were utilized in this investigation (CGSS). There are distinct differences in the effects based on gender, geographic location, and urban versus rural settings.

The following is a list of the findings from the study: (1) The subjective well-being of people living in China can be significantly improved through participation in physical exercise. (2) The subjective well-being of people living in China is affected in various ways by a variety of factors, including age, marital status, and political standing, among others. (3) Increasing the intensity of one’s physical exercise has been shown to have a greater impact on one’s subjective well-being in populations that are predominantly female, urban, and central and eastern.

## 6. Implications and Limitations

Previous research has focused on how physical exercise reduces negative emotions, but not how it affects positive emotions. They also tend to discuss the effects of physical exercise on mental health using only frequency. This study has theoretical significance because it integrates exercise frequency and intensity to explain the effect of exercise on Chinese residents’ subjective well-being. Second, the decline in subjective well-being is a global public health problem that threatens human health. This study can provide a way to improve people’s subjective well-being through physical exercise, which has social significance.

Future research should address this study’s limitations. First, because the literature on the heterogeneity of physical exercise’s impact on Chinese residents’ subjective well-being is limited, differences in exercise frequency, category, and time must be investigated. Second, in-depth research should be conducted on how exercise affects subjective well-being.

This study used secondary data, so measurement items were limited and all variables came from the same database, which could lead to homology bias. Future studies can use multi time series data from multiple sources to improve credibility. Cross-sectional data can only suggest a causal link between exercise and subjective well-being. Future group studies or additional data are recommended to assess the causal effects of physical exercise on Chinese residents’ subjective well-being.

## Figures and Tables

**Table 1:** Descriptive statistics.

Variable Types	Variable Name	Answer Options	M	SD	MIN	MAX
Explained variable	Subjective well-being	Very unhappy = 1, somewhat unhappy = 2, not really happy = 3, somewhat happy = 4, very happy = 5.	3.909	0.813	1	5
Core explanatory variable	Exercise frequency	Daily, several times a week, several times a month, several times a year or less = 1, never = 0.	0.576	0.494	0	1
	Exercise intensity	30 minutes of physical exercise per week that involves sweating	2.555	4.007	0	96
Individual level control variables	Gender	Male = 1, Female = 0.	0.512	0.500	0	1
	Age	Age of respondents	52.522	15.619	18	85
	Ethnicity	Han = 1, other = 0.	0.929	0.257	0	1
	Religion	No religious belief = 1, religious belief = 0	0.899	0.301	0	1
	Education	Years of education of respondents	8.918	4.721	0	19
	Income	The logarithm of the respondents’ annual income	9.942	1.344	0	16.113
	Party	Party member = 1, other = 0.	0.125	0.330	0	1
	Marriage	First marriage with a spouse, second marriage with a spouse, cohabitation = 1, other = 0.	0.805	0.397	0	1
	Household registration	Town = 1, rural = 0.	0.474	0.499	0	1
	Working hours	Weekly working hours of respondents	1.270	8.235	0	105
Household level control variables	Household income	The logarithm of the annual household income of the respondents	10.674	1.266	4.605	16.118
	Family size	Respondents’ household size	2.740	1.371	1	21
	Family SEs	Well below average = 1, below average = 2, average = 3, above average = 4, well above average = 5.	2.594	0.728	1	5
Social level control variables	Area	West = 0, central = 1, east = 2.	1.282	0.769	0	2
	Social	Never = 0, once a year or less = 1, several times a year = 2, about once a month = 3, several times a month = 4, once or twice a week = 5, every day = 6.	2.974	1.843	0	6
	SEs	Lower tier = 1, lower-middle tier = 2, middle tier = 3, upper-middle Tier = 4, upper tier = 5.	2.328	0.861	1	5
	Medicare	Yes = 1, no = 0.	0.935	0.247	0	1

**Table 2:** OLS regression results.

Variable	Model (1)	Model (2)	Model (3)	Model (4)
	Subjective Well-Being	Subjective Well-Being	Subjective Well-Being	Subjective Well-Being
Exercise frequency	0.143 ***	0.0779 ***	0.0629 ***	0.0400 **
	(0.0196)	(0.0210)	(0.0205)	(0.0203)
Exercise intensity	0.0127 ***	0.0107 ***	0.00947 ***	0.00913 ***
	(0.00242)	(0.00240)	(0.00234)	(0.00231)
Gender		−0.0619 ***	−0.0337 *	−0.0209
		(0.0179)	(0.0176)	(0.0174)
Age		0.00466 ***	0.00471 ***	0.00426 ***
		(0.000678)	(0.000681)	(0.000685)
Ethnicity		−0.0304	−0.0363	−0.0336
		(0.0350)	(0.0341)	(0.0336)
Religion		−0.0615 **	−0.0514 *	−0.0394
		(0.0298)	(0.0290)	(0.0286)
Education		0.00804 ***	0.00417 *	0.00304
		(0.00252)	(0.00247)	(0.00246)
Income		0.0704 ***	0.0140	0.00539
		(0.00858)	(0.0109)	(0.0108)
Party		0.107 ***	0.0676 **	0.0537 **
		(0.0284)	(0.0277)	(0.0273)
Marriage		0.165 ***	0.112 ***	0.112 ***
		(0.0220)	(0.0223)	(0.0221)
Household registration		−0.0183	−0.0256	−0.0351
		(0.0220)	(0.0216)	(0.0213)
Working hours		0.000112	−0.000138	−0.000125
		(0.00106)	(0.00103)	(0.00101)
Household income			0.0310 ***	0.0242 **
			(0.0115)	(0.0114)
Family size			0.0150 **	0.0146 **
			(0.00682)	(0.00675)
Family SEs			0.258 ***	0.137 ***
			(0.0127)	(0.0147)
Region				0.0163
				(0.0121)
Social				0.0167 ***
				(0.00467)
SEs				0.185 ***
				(0.0121)
Medicare				0.0374
				(0.0339)
Constant	3.794 ***	2.798 ***	2.396 ***	2.366 ***
	(0.0135)	(0.0993)	(0.109)	(0.112)
Observations	8445	8445	8445	8445
R2	0.016	0.045	0.096	0.123

Note: *** *p* < 0.01, ** *p* < 0.05, * *p* < 0.1, standard errors in parentheses.

**Table 3:** Grouping regression results.

Variable	Urban–Rural		Area			Gender	
	Rural	Urban	West	Middle	East	Female	Male
Exercise Frequency	0.0370	0.0486 *	0.0671	0.00871	0.0489 *	0.0365	0.0484 *
	(0.0284)	(0.0293)	(0.0471)	(0.0358)	(0.0292)	(0.0291)	(0.0286)
Exercise Intensity	0.00620 *	0.0116 ***	0.00402	0.00872 **	0.0136 ***	0.0122 ***	0.00538
	(0.00351)	(0.00302)	(0.00494)	(0.00411)	(0.00342)	(0.00314)	(0.00341)
Gender	0.00290	−0.0496 **	0.00187	−0.0112	−0.0416 *	-	-
	(0.0257)	(0.0236)	(0.0431)	(0.0317)	(0.0235)		
Age	0.00464 ***	0.00388 ***	0.00739 ***	0.00375 ***	0.00327 ***	0.00365 ***	0.00435 ***
	(0.00108)	(0.000891)	(0.00167)	(0.00131)	(0.000926)	(0.00102)	(0.000955)
Ethnicity	−0.0434	−0.00820	0.0375	−0.103	0.0284	−0.0200	−0.0446
	(0.0420)	(0.0591)	(0.0681)	(0.0692)	(0.0500)	(0.0475)	(0.0478)
Religion	−0.0283	−0.0529	−0.114 *	−0.0175	0.00139	−0.0315	−0.0480
	(0.0398)	(0.0412)	(0.0662)	(0.0579)	(0.0382)	(0.0368)	(0.0457)
Education	0.00748 **	−0.00210	0.0115 **	0.00276	−0.000501	−0.000480	0.00640 *
	(0.00365)	(0.00334)	(0.00570)	(0.00452)	(0.00342)	(0.00351)	(0.00351)
Income	−0.00539	0.0276	0.0453 *	−0.00361	−0.0156	−0.00157	0.00891
	(0.0141)	(0.0184)	(0.0233)	(0.0189)	(0.0163)	(0.0151)	(0.0158)
Party	0.0200	0.0761 **	0.0581	0.0344	0.0734 **	0.0880 *	0.0428
	(0.0562)	(0.0304)	(0.0833)	(0.0538)	(0.0333)	(0.0468)	(0.0340)
Marriage	0.113 ***	0.121 ***	0.0398	0.170 ***	0.124 ***	0.0841 ***	0.149 ***
	(0.0327)	(0.0299)	(0.0531)	(0.0422)	(0.0294)	(0.0311)	(0.0323)
Household registration	-	-	−0.0334	−0.0112	−0.0563 *	−0.0109	−0.0593 **
			(0.0524)	(0.0385)	(0.0297)	(0.0313)	(0.0294)
Working hours	−0.00176	0.00238	0.00375	−0.000559	−0.000978	−0.00201	0.00127
	(0.00137)	(0.00152)	(0.00285)	(0.00162)	(0.00145)	(0.00153)	(0.00136)
Household income	0.0301 **	0.0180	-0.00833	0.0506 ***	0.0123	0.0254	0.0259
	(0.0146)	(0.0188)	(0.0263)	(0.0195)	(0.0171)	(0.0156)	(0.0169)
Family size	0.0198 **	0.00657	0.0528 ***	−0.00396	0.00797	0.0158	0.0119
	(0.00902)	(0.0103)	(0.0154)	(0.0119)	(0.00975)	(0.00970)	(0.00943)
Family SEs	0.141 ***	0.129 ***	0.142 ***	0.131 ***	0.145 ***	0.161 ***	0.114 ***
	(0.0205)	(0.0212)	(0.0342)	(0.0272)	(0.0203)	(0.0215)	(0.0203)
Area	0.0286 *	−0.00393				0.0394 **	−0.00494
	(0.0165)	(0.0181)				(0.0176)	(0.0166)
Social	0.0111 *	0.0234 ***	0.00685	0.0177 **	0.0204 ***	0.0141 **	0.0180 ***
	(0.00638)	(0.00692)	(0.0115)	(0.00793)	(0.00674)	(0.00655)	(0.00672)
SEs	0.187 ***	0.178 ***	0.185 ***	0.183 ***	0.190 ***	0.173 ***	0.196 ***
	(0.0167)	(0.0176)	(0.0277)	(0.0224)	(0.0168)	(0.0175)	(0.0167)
Medicare	0.0518	0.0120	0.100	0.00345	0.0361	0.0769	0.00534
	(0.0447)	(0.0533)	(0.0810)	(0.0583)	(0.0489)	(0.0487)	(0.0474)
Constant	2.312 ***	2.310 ***	2.021 ***	2.291 ***	2.726 ***	2.370 ***	2.353 ***
	(0.158)	(0.188)	(0.260)	(0.213)	(0.165)	(0.161)	(0.159)
Observations	4446	3999	1640	2782	4023	4123	4322
R2	0.107	0.129	0.124	0.113	0.131	0.127	0.123

Note: *** *p* < 0.01, ** *p* < 0.05, * *p* < 0.1, standard errors in parentheses.

**Table 4:** Comparison of experimental and control groups after PSM.

Variable	Unmatched	Mean			%Reduct	t-Test		V(T)/
	Matched	Treated	Control	%Bias	|Bias|	t	*p* > t	V(C)
Gender	U	0.51623	0.50573	2.1		0.95	0.34	
	M	0.51623	0.50637	2	6.1	0.97	0.331	
Age	U	51.046	54.532	−22.6		−10.2	0	1.14 *
	M	51.046	52.271	−7.9	64.8	−3.79	0	1
Ethnicity	U	0.94967	0.9002	18.9		8.77	0	
	M	0.94967	0.9544	−1.8	90.5	−1.09	0.275	
Religion	U	0.90612	0.89013	5.3		2.41	0.016	
	M	0.90612	0.89359	4.1	21.6	2.06	0.039	
Education	U	10.301	7.0366	73.7		33.4	0	1.05
	M	10.301	10.162	3.1	95.8	1.55	0.12	1.09 *
Income	U	10.324	9.4229	70.2		32.25	0	0.75 *
	M	10.324	10.311	1	98.6	0.53	0.597	0.98
Party	U	0.16311	0.07213	28.5		12.63	0	
	M	0.16311	0.15715	1.9	93.5	0.8	0.423	
Marriage	U	0.79951	0.81157	−3		−1.38	0.167	
	M	0.79951	0.78554	3.5	−15.8	1.7	0.089	
Household registration	U	0.61031	0.28739	68.6		30.99	0	
	M	0.61031	0.62654	−3.4	95	−1.65	0.099	
Working hours	U	1.3014	1.2276	0.9		0.41	0.684	0.85*
	M	1.3014	1.1561	1.8	−96.8	0.92	0.356	1.10 *
Household income	U	11.031	10.187	69.9		32.04	0	0.77 *
	M	11.031	11.007	2	97.1	1.06	0.291	1
Family size	U	2.7124	2.7786	−4.8		−2.19	0.028	0.90 *
	M	2.7124	2.7182	−0.4	91.3	−0.22	0.83	1.07 *
Family SEs	U	2.6972	2.4526	33.9		15.47	0	0.90 *
	M	2.6972	2.7062	−1.3	96.3	−0.64	0.525	1
Region	U	1.4141	1.1026	41.1		18.78	0	0.85 *
	M	1.4141	1.3969	2.3	94.5	1.15	0.248	0.94 *
Social	U	3.2479	2.6005	35.3		16.2	0	0.74 *
	M	3.2479	3.2734	−1.4	96.1	−0.71	0.479	0.84 *
SEs	U	2.4577	2.1521	35.9		16.37	0	0.89 *
	M	2.4577	2.4706	−1.5	95.8	−0.75	0.45	0.91 *
Medicare	U	0.94269	0.92396	7.5		3.45	0.001	
	M	0.94269	0.94988	−2.9	61.6	−1.57	0.116	

Note: * *p* < 0.1.

**Table 5:** Matching effect test.

Method	Pseudo R^2^		LR chi2		*p* > chi2	
	Unmatched	Matched	Unmatched	Matched	Unmatched	Matched
K-nearest neighbor matching (k = 1)	0.095	0.003	1113.940	37.520	0.000	0.906
K-nearest neighbor matching (k = 4)	0.095	0.002	1113.940	24.980	0.000	0.999
Radius matching (0.05)	0.095	0.002	1113.940	25.780	0.000	0.999
Kernel matching	0.095	0.002	1113.940	23.530	0.000	0.997

## Data Availability

The data of CGSS2018 are publicly available at http://cgss.ruc.edu.cn (accessed on 30 December 2021).
